# Clinical feasibility of primary retrograde endovascular therapy via trans-ankle intervention: a retrospective single-center study

**DOI:** 10.1186/s42155-026-00735-2

**Published:** 2026-07-16

**Authors:** Hiromi Miwa, Naoki Hayakawa, Toshiki Tsurumaki, Yasuyuki Tsuchida, Masanao Inoue, Shinya Ichihara, Shunichi Kushida

**Affiliations:** Department of Cardiovascular Medicine, Asahi General Hospital, Chiba, Japan

**Keywords:** Endovascular therapy, Femoropopliteal artery, Tibial artery, Trans-ankle intervention

## Abstract

**Background:**

Trans-ankle intervention (TAI) through retrograde distal access via below-the-knee and below-the-ankle arteries has been increasingly reported as an alternative approach for the treatment of femoropopliteal (FP) lesions. Although previous studies have described the procedural success of this strategy, its clinical feasibility and post-procedural outcomes remain insufficiently characterized.

**Methods:**

In this retrospective single-center study, the data from 33 patients with 36 FP lesions who underwent endovascular therapy (EVT) between January 2024 and January 2025 were analyzed. The primary endpoint was procedural success. The secondary endpoints were clinical worsening of lower-limb ischemia within 30 days, 1-year freedom from clinically driven target lesion revascularization (CD-TLR), and procedural complications. The risk of post-procedure access vessel occlusion was also investigated.

**Results:**

TAI was performed via access sites ranging from the anterior tibial artery (ATA) to the dorsalis pedis artery in all patients. Procedural success was achieved in all cases, and the rate of 1-year freedom from CD-TLR was 91.8%. All patients had FP lesions, including three cases up to the iliac artery. Forty-two percent was chronic total occlusion, and EVT of the ATA was required in 42% of the cases to establish the access route. Ten cases (27.8%) were approached via the occluded pre-ATA, and 15 (41.7%) required intervention to approach the vessel. The average number of pre-procedural and post-procedural below-the-knee artery runoff vessels was 2.4 and 2.75, respectively. No patients showed clinical worsening within 30 days. The risk factors for ATA occlusion were hemodialysis, ATA intervention, and approach via the occluded ATA.

**Conclusions:**

TAI may represent a feasible result, especially for selected FP lesions. Although no clinical worsening was observed, an increased risk of postoperative ATA occlusion existed in patients on hemodialysis and those with access site atherosclerosis requiring ATA intervention. However, in these high-risk populations, this limitation may potentially be overcome by an approach through the occluded ATA.

## Background

Endovascular therapy (EVT) has become a standard treatment for patients with peripheral artery disease (PAD) [1]. There are several approaches for femoropopliteal (FP) artery revascularization. The transfemoral approach (TFA) is the most commonly used. Retrograde tibioperoneal access is a safe option for EVT after antegrade approach failure [2, 3]. However, it is rare to complete this procedure using a primary retrograde approach. In percutaneous coronary intervention (PCI), one study suggested that compared with the TFA, the transradial approach (TRA) showed reduced mortality and major adverse cardiovascular event rates, as well as improved safety, with reductions in major bleeding and vascular complications [4]. The efficacy and safety of the TRA for PCI have been well established, making it a standard access site. In EVT procedures, the TRA has demonstrated non-inferiority to the TFA, allowing less invasive treatment [5]. However, its application is limited to the treatment of the iliac arteries or the proximal portion of the superficial femoral artery due to the effective length of each device.

In recent years, the development of lower-profile guiding sheaths has enabled the use of 6-Fr-equivalent guiding sheaths, even via the tibial arteries. In this context, trans-ankle intervention (TAI) has emerged as a viable minimally invasive treatment option for FP lesions via dorsalis pedis artery (DPA) or anterior tibial artery (ATA) access. TAI has been reported as a successful and effective technique for the treatment of patients with PAD [6]. Using a combination of tibiopedal access and the TRA, a previous study showed a procedural success rate of 99%, even in patients with FP chronic total occlusion (CTO) [7]. However, the clinical course of patients following the use of tibial or other below-the-knee (BTK) arteries as access sites has not been sufficiently reported.

However, tibial arteries in patients with PAD are frequently affected by diffuse atherosclerotic disease. Therefore, the favorable outcomes reported for TRA in PCI cannot be directly extrapolated to TAI. In the present study, we evaluated the efficacy and safety of TAI, with particular focus on patient symptoms and the clinical course of the access site.

## Methods

### Ethics

The study protocol was approved by the ethics committee of Asahi General Hospital, and the study was performed in accordance with the Declaration of Helsinki. The requirement for informed consent was waived because of the retrospective study design, in which existing medical records were used to obtain patient data. However, patients were given the option to opt out of the study. Relevant information regarding the study is available to the public in accordance with the Ethical Guidelines for Medical and Health Research Involving Human Subjects.

### Patients and study design

**Fig. 1:**
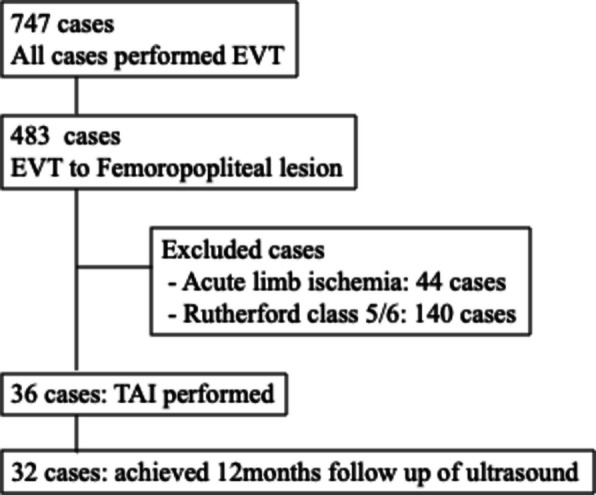
Study flowchart. EVT, endovascular therapy; TAI, trans-ankle intervention

In this retrospective, single-center cohort study, the data of consecutive patients diagnosed with PAD at Asahi General Hospital between January 2024 and January 2025 were analyzed, including 36 patients who underwent primary TAI for FP lesions (Fig. 1). Patients with chronic limb-threatening ischemia (CLTI) and acute limb ischemia were excluded because these conditions are contraindications for TAI. The choice of TAI was determined at the discretion of each operator. Patients who were preferentially considered for enrollment in TAI were as follows: prior access site complication, obesity, difficulty in cross-over approach and difficulty in maintaining rest time. Lesion morphology was primarily assessed before the procedure using CT angiography or duplex ultrasound. Patients were excluded if TAI was considered unfeasible during pre-procedural assessment, defined as the absence of a DPA and an ATA diameter < 2 mm on ultrasound, or unsuitability as judged by the operator. In addition, cases in which the ATA represented the only BTK runoff vessel were considered unsuitable for TAI because ATA occlusion could potentially worsen limb ischemia and compromise future distal bypass options. In cases where both the ATA and PTA were occluded, access was obtained through the occluded ATA with the additional aim of improving BTK runoff.

### EVT procedure

Dual antiplatelet therapy, defined as the administration of aspirin and clopidogrel or prasugrel, or antiplatelet and anticoagulant therapy, was used at least 1 month after the procedure. At least one antiplatelet or anticoagulant drug was administered for a minimum of 6 months after the EVT procedure.

A 6-Fr guiding sheath (Parent Select 5082®; Medikit, Japan) or a 7-Fr guiding sheath (Parent Pro 60®; Medikit) was inserted into the ipsilateral ATA via the DPA. In our institution, ATA was selected in all cases because ultrasound-guided puncture was technically easier. The puncture site and type of guiding sheath were determined by the ultrasound findings. Patients with a vessel diameter smaller than 2 mm were excluded, however, precise measurements of the vessel diameters were not recorded. After guiding sheath insertion, an intravenous heparin bolus (5000 units) was administered, followed by an infusion of 1000 units per hour. After 0.014-, 0.018-, and 0.035-inch guidewires were passed through the ATA, the guiding sheath was inserted via the ATA. If the ATA was occluded or showed very severe, diffusely calcified stenosis that prevented sheath insertion, ATA intervention was performed to insert the guiding sheath prior to FP treatment. Plain old balloon angioplasty (POBA) of the ATA was performed using a 2.5-mm or 3.0-mm balloon. Intravascular ultrasound (IVUS) was used for lesion assessment, if needed. When guidewire passage through the occluded ATA was challenging, IVUS-guided wiring was performed [8]. The ATA was then used as an access route after successful guidewire advancement into the proximal true lumen.

The guiding sheath was advanced to the popliteal artery, and angiography was performed. When retrograde angiography did not allow adequate assessment of the entire FP segment, a 4-Fr catheter or an aspiration catheter was advanced to the common femoral artery (CFA) after guidewire passage, followed by angiography. Then, 0.014-, 0.018-, or 0.035-inch guidewires were used with a microcatheter or backup support catheter. A bidirectional approach was used if the conventional single-directional guidewire crossing failed. In CTO lesions especially extending to the SFA proximal, when necessary, an antegrade sheath or the outer sheath of the needle was additionally used. The bidirectional approach was performed via the ipsilateral CFA, the contralateral CFA, or the TRA. After guidewire passage, pre-dilation was performed using an optimally sized balloon (IVUS- or quantitative vascular angiography-based). The type of balloon (semi-compliant, non-compliant, cutting, or scoring) was at each clinician’s discretion. The drug-coated balloon (DCB) was used after confirming that residual stenosis was < 50% and the degree of dissection was less than grade D based on the National Heart, Lung and Blood Institute (NHLBI) criteria [9]. Pressure gradient evaluation was performed as required, and pressure gradients < 10 mmHg were defined as significant stenosis [10]. After successful lesion preparation, the target lesion was fully covered by the DCB. If > 50% residual stenosis or NHLBI grade D or higher dissection was observed after using the DCB, bailout stenting was considered. The necessity for stent use was judged by each clinician. Atherectomy devices require compatibility with a 7-Fr or larger guiding sheath. Therefore, their use was considered only in cases where a 7-Fr guiding sheath could be inserted based on the vessel diameter assessed by preprocedural ultrasound findings. After completion of the FP intervention, angiography of the BTK was performed while withdrawing the sheath to evaluate blood flow of the BTK and access vessel-related complications. Hemostasis of the DPA was achieved using a tape (Astepty®; Nichiban, Japan) or a hemostasis band (Bleed Safe®; Medikit, Japan). There was no completely standardized protocol for hemostasis; however, a general approach was followed in clinical practice. For Astepty, hemostasis was assessed approximately 2 h after fixation, and additional fixation was applied if rebleeding occurred. For Bleed Safe, compression was initiated with ≥ 10 mL pressure and gradually reduced by 2 mL every hour after hemostasis was achieved, with re-compression applied in cases of rebleeding. Acute access site complications were defined as composite of puncture site hematoma, pseudoaneurysm, arteriovenous fistula, and overt puncture site bleeding on the first day after procedure [11]. Patients were allowed free ambulation after the procedure. They were observed overnight and discharged the following morning. If a stent was placed, at least two antithrombotic drugs were administered for at least 1 month.

### Data definitions

Among the patients with PAD, patients with lower-extremity artery disease were included. Severity was assessed using the Rutherford classification [12] at the outpatient visit prior to hospitalization. CLTI was defined as a Rutherford class of 4–6. However, for convenience, Rutherford classes 5 and 6 were denoted as CLTI in this study. Clinical worsening included worsening of claudication or pain at rest and formation of new wounds. Ambulatory status was assessed at the time of hospitalization, and the non-ambulatory group included patients using wheelchairs. The status of the ATA was classified as either occluded or open, with stenosis included in the open category. It was evaluated separately for pre-procedural ATA (pre-ATA) and post-procedural ATA (post-ATA). Pre-ATA status was determined based on ultrasound examination, computed tomography (CT), and angiographic findings. Follow-up of the target lesion and post-ATA status was performed by ultrasound examination or CT. Follow-up of the puncture site was performed by ultrasound, Doppler examination, palpation of the DPA, or CT. The modality and timing of follow-up were determined at the discretion of each doctor, and follow-up assessments were performed during the chronic phase.

### Outcomes

The primary outcome was procedural success. The secondary outcomes included clinical worsening of lower-limb ischemia within 30 days, 1-year clinically driven target lesion revascularization (CD-TLR), and procedural complications. Procedural success was defined as successful recanalization of the target lesion with < 30% residual stenosis on final angiography. Clinical follow-up was performed 30 days after EVT with a tolerance of ± 2 months, and 12 months after EVT with a tolerance of ± 2 months. Clinical events were evaluated by at least two specialists from the Japanese Association of Cardiovascular Intervention and Therapeutics.

### Statistical analysis

Statistical analyses were performed using Stata, version 16 (StataCorp, College Station, TX, USA). Normally distributed data were presented as the mean ± standard deviation, while non-normally distributed data were presented as the median (interquartile range). Categorical data are presented as number (percentage). *P* < 0.05 was considered statistically significant, and 95% confidence intervals (CIs) are reported where appropriate. Based on the hypothesis that post-ATA occlusion contributes to clinical outcomes, we investigated post-ATA occlusion and its risk factors. The association was investigated using a logistic regression model. Univariate analysis was performed using the following factors: ambulant, hemodialysis, coronary artery disease, Rutherford ≥ 4, PACSS grade ≥ 3, approach from occluded ATA, intervention to approach ATA.

## Results

### Patients’ demographic and clinical characteristics

Overall, 33 patients underwent EVT and were included in the analysis. Three patients underwent bilateral limb EVT. The patients’ characteristics are summarized in Table 1. The median age was 75 years. Thirty-one cases (86.1%) were ambulatory and nine (25%) were on hemodialysis. Most of the patients had intermittent claudication, and three cases (8.3%) were classified as Rutherford class 4. In 34 cases (94.4%), the FP artery was the target lesion, and CTO lesions accounted for 15 cases (42.0%).

**Table 1:** Patients’ demographic and clinical characteristics

Characteristics	Number	
Male	33	91.7%
Right	21	58.3%
Age, median [IQR]	75 [70–81.25]	
Body mass index	23.3 ± 3.72	
Ambulatory	31	86.1%
Hypertension	32	88.9%
Diabetes mellitus	21	58.3%
Dyslipidemia	35	97.2%
Smoke history		
Never smoker	2	5.6%
Past smoker	22	61.1%
Current smoker	11	30.6%
Kidney function		
Normal	17	47.2%
Chronic kidney disease without hemodialysis	10	27.8%
Hemodialysis	9	25%
Stroke	8	22.2%
Coronary artery disease	21	58.3%
Congestive heart failure	10	27.8%
Atrial fibrillization	5	13.9%
Aortic stenosis > moderate	1	4.8%
Rutherford classification		
2	17	47.2%
3	16	44.4%
4	3	8.3%
Medication		
Aspirin	32	88.9%
P2Y12 inhibitor	31	86.1%
Cilostazol	5	13.9%
Anticoagulant	5	13.9%
Statin	26	72.2%
Lesion location		
Iliac artery	3	8.3%
Femoral artery	34	94.4%
Popliteal artery	4	11.1%
Chronic total occlusion	15	42.0%
Denovo	24	66.7%
Calcium location	19	52.8%
PACSS grade		
0	10	27.8%
1	4	11.1%
2	3	8.3%
3	7	19.4%
4	12	33.3%

*IQR* interquartile range, *PACSS* peripheral artery calcium scoring system

### Procedural characteristics

The revascularization procedure characteristics are shown in Table 2. Procedural success was achieved in all cases (100%). Ten cases (27.8%) were approached via the occluded pre-ATA, and 15 (41.7%) required intervention to approach the vessel. Eight cases (22.2%) required an additional antegrade approach, and four of these required antegrade access only for angiographic evaluation. Among the remaining four cases requiring both angiography and antegrade intervention, three involved CTO extending from the proximal SFA, in which assessment of the CFA bifurcation was necessary. The other case involved thrombotic occlusion requiring thrombectomy with an 8-Fr sheath, and additional antegrade sheath was needed. The average number of pre-procedural and post-procedural BTK run-off vessels was 2.4 and 2.75, respectively. The median procedure time was 54 min. Slow flow was not observed in any case, while distal embolization was observed in three cases (8.3%). Puncture site complication occurred in three cases (8.3%), all of which were minor hematomas at the puncture site.

**Table 2:** Procedural characteristics

Characteristics		
Procedural success	36	100%
Approach from occluded ATA	10	27.8%
Intervention to approach ATA	15	41.7%
Pre-BTK run off (number)	average	2.4
0, 1	6	17.1%
2, 3	29	82.9%
Post-BTK run off (number)	average	2.75
0, 1	1	2.78%
2, 3	35	97.2%
Post- approach ATA run off	36	100%
Additional antegrade approach (only angiography)	8 (4)	22.2%
Procedure time, min, median [IQR]	54 [16–85]	
Radiation exposure (TKA) mGy	135 [11.5–216.5]	
Finalize device		
Stent	2	5.6%
Hybrid therapy	2	5.6%
Drug coated balloon	32	88.9%
Balloon	1	2.8%
Final NHLBI dissection		
0, a	28	77.8%
b	7	19.4%
c	1	2.8%
d	0	0%
Bail-out stenting	0	0%
Jetstream	1	2.8%
Crosser	5	13.9%
Wingman	9	25.0%
Fracking	6	17.1%
Intravascular ultrasound use	11	30.6%
Slow flow	0	0%
Distal embolization	3	8.3%
Procedure related complication	0	0%
Puncture site complication	3	8.3%

*ATA* anterior tibial artery, *BTK* below the knee, *IQR* interquartile range, *TKA* total air karma, *NHLBI* National Heart, Lung and Blood Institute

### Patient outcomes

**Fig. 2:**
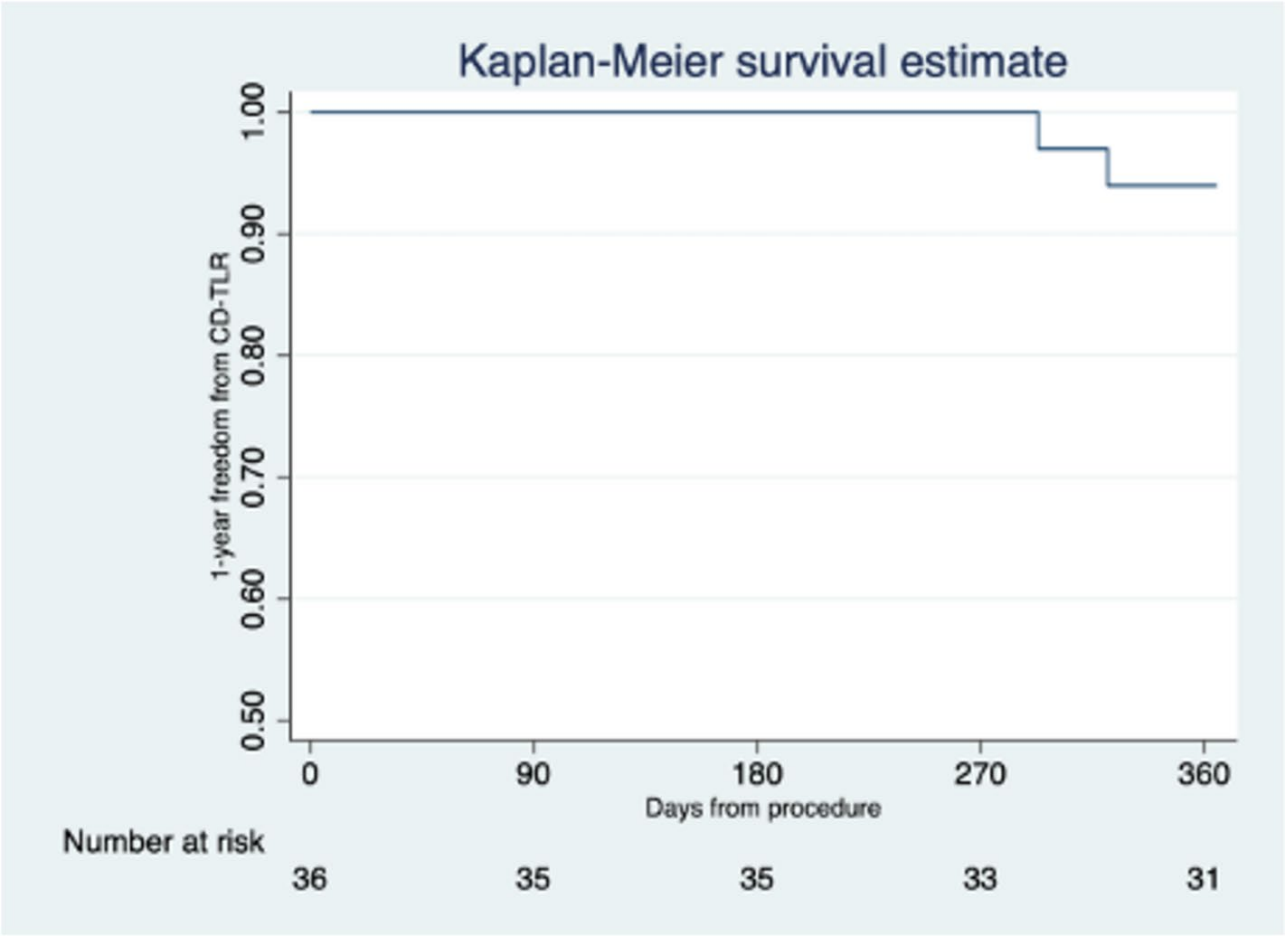
Kaplan–Meier curves of freedom from CD-TLR. CD-TLR, clinically driven target lesion revascularization

The clinical outcomes of the patients are shown in Table 3. No patients showed clinical worsening of lower-limb ischemia within 30 days. The 1-year CD-TLR was 91.8% (Fig. 2). Access vessel follow-up was performed using CT or ultrasound, and seven cases (21.9%) experienced occlusion. Follow-up assessment of ATA status was achieved for a median duration of 307 days after the procedure. Puncture site assessment in the acute phase was performed during hospitalization and at the subsequent outpatient clinic visit.

**Table 3:** Clinical outcomes of the patients

Characteristic		
Mortality (1 year)	2	5.6%
Amputation (1 year)	0	0%
Clinical worsening (within 30 days)	0	0%
Clinical worsening (within 1 year)	1	2.9%
Freedom from CD-TLR (within 1 year)	31	91.8%
Access vessels (post-procedural ATA) follow up Achieved in 32 cases		
Open	25	78.1%
Occluded	7	21.9%
Puncture sites (post-procedural DPA) follow up Achieved in 36 cases		
Open	34	94.4%
Occluded	2	5.6%

*ATA* anterior tibial artery, *CD-TLR* clinically driven target lesion revascularization; *DPA* dorsalis pedis artery

### Risk factors for post-procedural ATA occlusion

The risk factors for post-ATA occlusion were evaluated (Table 4). Hemodialysis, pre-ATA status, and ATA intervention were significant risk factors for post-ATA occlusion.

**Table 4:** Risk factors of post-procedural ATA occlusion

	OR [95% CI]	*p* value
Ambulant	0.52 [0.04–6.77]	0.62
Hemodialysis	28.8 [3.23–255.8]	0.003
Coronary artery disease	2.31 [0.37–14.2]	0.37
Rutherford ≥ 4	1.92 [0.15–24.9]	0.62
PACSS grade ≥ 3	2.71 [0.44–16.7]	0.28
Approach from occluded ATA	10.0 [1.48–67.6]	0.018
Intervention to approach ATA	–	< 0.001

*ATA* anterior tibial artery, *CI* confidence interval, *OR* odds ratio, *PACSS* peripheral artery calcium scoring system

**Fig. 3:**
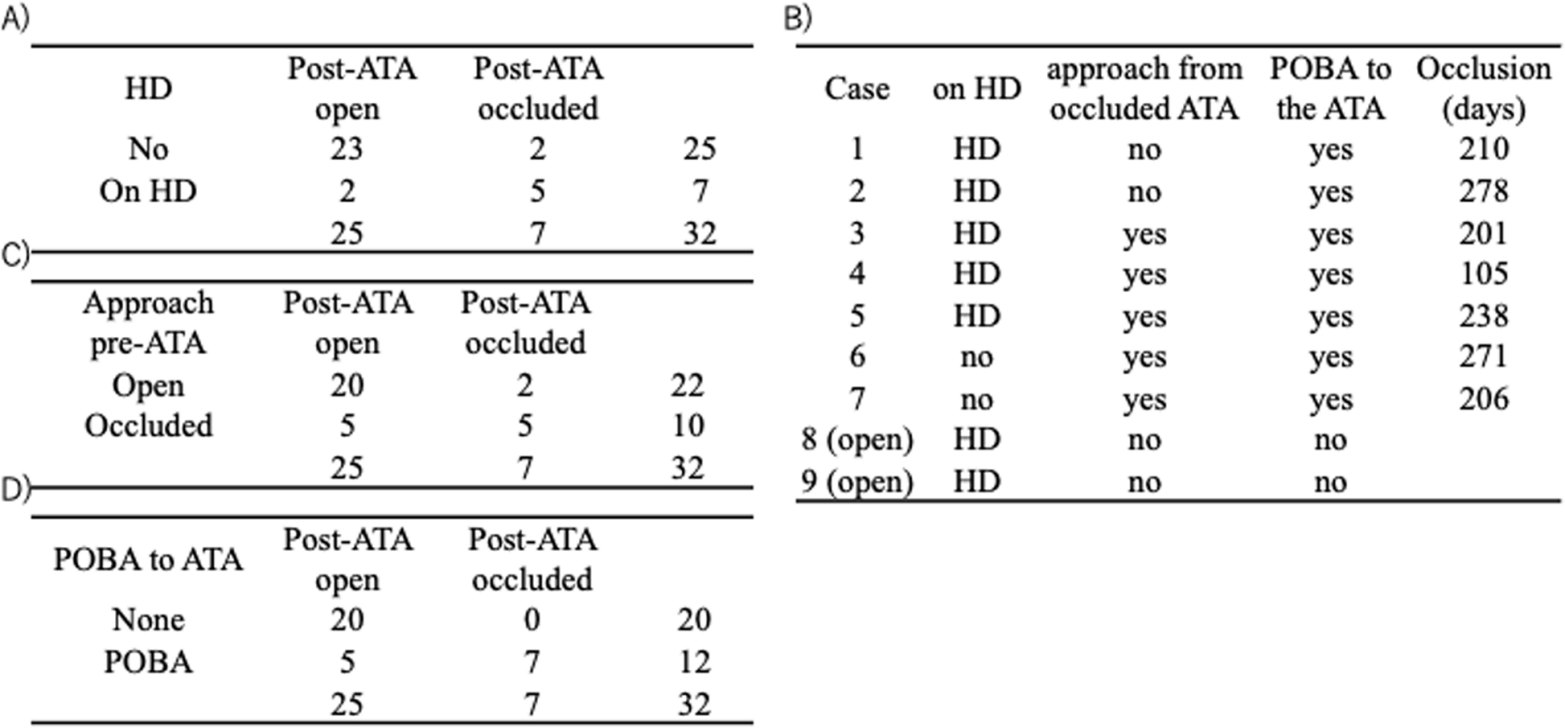
Distribution of risk factors for post-procedural ATA occlusion. Hemodialysis (**a**), pre-procedural ATA status (**c**), and ATA intervention (**d**) were significant risk factors for post-procedural ATA occlusion. **b** All post-procedural ATA occlusion cases (patients 1–7) and all patients on hemodialysis are shown. The days to the first detection of ATA occlusion were shown. Patients 8 and 9 were on hemodialysis, and post-procedural ATA patency was maintained. HD, hemodialysis; ATA, anterior tibial artery; POBA, plain old balloon angioplasty

The distribution of risk factors for post-procedural ATA occlusion is shown in Fig. 3. Among patients on hemodialysis, post-ATA occlusion occurred in five of seven (71.4%) (Fig. 3A). All post-procedural ATA occlusion cases (patients 1–7) and all patients on hemodialysis are shown (Fig. 3B). Patients 1 and 2 had patent pre-ATA lesions but required POBA and subsequently developed occlusion. Patients 8 and 9 were on hemodialysis, and post-procedural ATA patency was maintained. All of these patients underwent ATA POBA. In contrast, the two patients in whom post-ATA occlusion did not occur were approached via the patent pre-ATA and did not undergo ATA POBA. The open pre-ATA approach demonstrated two cases of occlusion, both involving patients on hemodialysis requiring ATA POBA (Fig. 3C). Meanwhile, the occluded pre-ATA approach-maintained patency in half of the patients postoperatively. For patients in whom ATA POBA was not performed, post-ATA remained patent in all patients (Fig. 3D). When POBA was performed, the post-ATA became occluded in 7 of 12 cases; all of these were either patients on hemodialysis or patients in whom the occluded pre-ATA was used as the approach site.

## Discussion

In this study, procedural success was achieved in all patients, and the 1-year freedom from CD-TLR was 91.8%, showing feasible results compared with a previous FP treatment study [13]. TAI was performed in a highly selected patient population; among 299 cases undergoing EVT for FP lesions, 36 cases were selected for the TAI approach. However, in this study, 42% of the patients had CTO of the FP lesion, suggesting that this approach site may be technically feasible even for treating complex lesions. The procedure time, including access route establishment, was 54 min, which was considered acceptable. The high success rate of this procedure is believed to have been achieved because the treatment options were not limited and the 6-Fr guiding sheath was inserted. Stent deployment, insertion of specific systems, and IVUS-guided parallel wiring were performed with less difficulty. Also, combination with extra-vascular ultrasound-guided may be useful for SFA EVT. Debulking device insertion was challenging as it required a 7-Fr guiding sheath. In the single case requiring a 7-Fr system, ipsilateral access was considered to carry a relatively high risk because of obesity, severe calcification at the puncture site, and a previous complication. In contrast, ultrasound evaluation demonstrated a relatively large distal vessel diameter of approximately 3 mm, and therefore TAI was selected as the safer and more feasible approach in this case. This case achieved success, with favorable clinical outcomes, persistent post-ATA patency and the 1-year CD-TLR rate was also acceptable. This finding suggests that, even with TAI, there were minimal device-related limitations in FP treatment, potentially allowing intervention with comparable quality to that achieved via the TFA. In fact, a 6-Fr guiding sheath (or larger) was successfully inserted in all cases, enabling treatment with virtually no device restrictions, such as DCBs and stents.

The insertion of balloons and stents is possible up to the ipsilateral iliac artery. As demonstrated by the inclusion of three patients with treatment of the iliac artery in this study, treatment from the ipsilateral iliac artery to BTK arteries is feasible with TAI. However, as TAI is retrograde, it has the disadvantage of difficulty in angiography. For occlusive FP lesions, an antegrade approach was used when necessary. Antegrade sheath insertion was performed in eight patients, with four of these used solely for angiography. Combining this with the TRA enabled a minimally invasive procedure, avoiding femoral artery puncture. The high success rate of unidirectional wiring, even in CTO procedures, was likely attributable to the adjunctive use of IVUS-guided wiring.

Procedure-related complications included distal embolism in 3 cases (8.3%). All cases involved distal embolization associated with SFA balloon dilation, and the clinical impact appeared to be minimal. Of the three cases, two involved CTO and one involved a severely calcified lesion. Therefore, lesion complexity itself may have contributed to the higher risk of distal embolization. In all cases, bailout treatment was successfully achieved with aspiration.

These complications do not appear to be specific to the TAI procedure itself. However, one possible contributing factor may be the maneuver of pulling the balloon toward the distal side. In addition, the excellent angiographic visualization may have contributed to easier detection and diagnosis of distal embolization. No bleeding complications, including those related to the access site, or cerebrovascular events, such as stroke, were recorded. These findings suggest the feasibility of TAI; however, potential risks such as access vessel injury should be carefully considered.

No patients demonstrated clinical worsening of lower-limb ischemia within 30 days, suggesting that this access site is feasible. However, asymptomatic worsening could not be detected. At the timing of case selection, BTK runoff was evaluated. If the ATA was the only BTK runoff vessel, TAI was considered unfavorable because the risk of ATA occlusion remained unclear and ATA occlusion could potentially worsen limb ischemia and compromise future bypass options. However, this risk could be reduced in cases with preserved PTA flow. Furthermore, using an occluded ATA as the access route may further mitigate this risk.

Although ATA access was selected in all cases in the present study because puncture and hemostasis were considered technically straightforward, PTA access may be preferable in selected patients. PTA access may be considered when preservation of ATA patency is important, such as in patients with ATA-dependent runoff or when the ATA may be considered as a future distal bypass target. Anatomically, PTA access may be more suitable when the distal PTA has a relatively straight course and is considered favorable for puncture and sheath insertion. Furthermore, improvements in dedicated hemostatic devices may stimulate broader use of PTA access in the future.

Therefore, we assessed the risk of post-ATA occlusion, which could lead to lower-limb ischemia. Hemodialysis, pre-ATA status, and ATA intervention were significant risk factors for post-ATA occlusion. Patients on hemodialysis and patients with severe ATA atherosclerosis who require POBA for guiding sheath insertion potentially carry a risk of further atherosclerotic progression. Although the possibility of natural progression remains, the contribution of iatrogenic injury cannot be ruled out, and the appropriateness of TAI as an access site should be carefully considered. In patients on hemodialysis undergoing PCI, the TRA shows a lower risk of access site bleeding and in-hospital mortality than the TFA, suggesting that the radial artery is a safe access site [14, 15]. This finding likely reflects the underlying clinical background of patients on hemodialysis, who are at an increased risk of bleeding and are more prone to access site complications. TAI carries a risk of occlusion due to its approach via small-diameter vessels, but it holds potential for reducing bleeding complications.

If the pre-ATA is occluded, there is a risk of reocclusion. This is because it is difficult to maintain patency after BTK artery treatment [16]. However, post-ATA reocclusion is unlikely to be a significant problem. This is because when an ATA that was already occluded is used as an access route, any subsequent reocclusion would simply return the vessel to its pre-procedural state. Therefore, using the occluded pre-ATA as an access site is reasonable. Using the TRA for PCI, studies have also demonstrated the utility of trans-occluded radial access [17]. Although it is technically challenging, it is a feasible option, even for patients with CLTI [18]. IVUS-guided wiring is an effective technique for BTK CTO treatment [8]. Even in patients with unstable hemodynamics and limited access sites, hemostatic procedures via the occluded ATA can be performed safely using IVUS-guided wiring [19].

However, TAI in patients with CLTI remains controversial. One study showed that below-the-ankle distal puncture in stenotic arteries may cause puncture site occlusion before wound healing [20]. The outer diameter of the sheath used in this study was 2.4 mm, suggesting the risk of puncture site occlusion if the DPA was small. Another study showed that using the approach from a single remaining BTK vessel showed feasible and safe results, and access vessel occlusion occurred in 9 of 314 patients [21]. The application of TAI in patients with CLTI should be carefully considered.

This study had several limitations. It was a retrospective, non-randomized, single-center study with a small sample size, and was not designed for comparative evaluation; therefore, the results may not be generalizable. The choice of access strategy, the timing of conversion to the bidirectional approach, and device selection were at the operator’s discretion, and no standardized protocol was used. In our institution, TFA remains a standard approach, and patient selection was based on the operator’s judgment of TAI feasibility, which may have introduced selection bias. Therefore, the favorable outcomes observed in this study should be interpreted with caution due to the highly selected nature of the study population. Furthermore, all angiographic findings and clinical outcomes were assessed on site, without adjudication by an independent core laboratory or clinical events committee. Therefore, future large-scale prospective studies are warranted to validate our findings.

## Conclusions

TAI may represent a feasible EVT approach for selected FP lesions. Although no clinical worsening was observed, the risk of post-ATA occlusion was increased in patients on hemodialysis and access site atherosclerotic pre-ATA requiring POBA. However, this limitation may potentially be surmounted by approaching from the occluded pre-ATA.

## Data Availability

The datasets used and/or analyzed during the current study are available from the corresponding author on reasonable request.

## Abbreviations

ATA: Anterior tibial artery
BTK: Below-the-knee
CD-TLR: Clinically driven target lesion revascularization
CFA: Common femoral artery
CI: Confidence interval
CLTI: Chronic limb-threatening ischemia
CT: Computed tomography
CTO: Chronic total occlusion
DAPT: Dual antipatelet therapy
DCB: Drug-coated balloon
DPA: Dorsal pedalis artery
EVT: Endovascular therapy
FP: Femoropopliteal
GS: Guiding sheath
IVUS: Intravascular ultrasound
NHLBI: National Heart, Lung and Blood Institute
PACSS: Peripheral artery calcium scoring system
PAD: Peripheral artery disease
PCI: Percutaneous coronary intervention
POBA: Plain old balloon angioplasty
Post-ATA: Post-procedural anterior tibial artery
Pre-ATA: Pre-procedural anterior tibial artery
TAI: Trans-ankle intervention
TFA: Transfemoral approach
TRA: Transradial approach

## References

[1] Gornik HL, Aronow HD, Goodney PP, Arya S, Brewster LP, Byrd L, et al. 2024 ACC/AHA/AACVPR/APMA/ABC/SCAI/SVM/SVN/SVS/SIR/VESS guideline for the management of lower extremity peripheral artery disease: a report of the American College of Cardiology/American Heart Association Joint Committee on Clinical Practice Guidelines. Circulation. 2024;149(24):e1313–410. 38743805 10.1161/CIR.0000000000001251 PMC12782132

[2] Schmidt A, Bausback Y, Piorkowski M, Wittig T, Banning-Eichenseer U, Thiele H, et al. Retrograde tibioperoneal access for complex infrainguinal occlusions: short- and long-term outcomes of 554 endovascular interventions. JACC Cardiovasc Interv. 2019;12(17):1714–26. 31488299 10.1016/j.jcin.2019.06.048

[3] Giannopoulos S, Palena LM, Armstrong EJ. Technical success and complication rates of retrograde arterial access for endovascular therapy for critical limb ischaemia: a systematic review and meta-analysis. Eur J Vasc Endovasc Surg. 2021;61(2):270–9. 33358346 10.1016/j.ejvs.2020.11.020

[4] Ferrante G, Rao SV, Jüni P, Da Costa BR, Reimers B, Condorelli G, et al. Radial versus femoral access for coronary interventions across the entire spectrum of patients with coronary artery disease: a meta-analysis of randomized trials. JACC Cardiovasc Interv. 2016;9(14):1419–34. 27372195 10.1016/j.jcin.2016.04.014

[5] Iida O, Takahara M, Fujihara M, Higashino N, Hayakawa N, Horie K, et al. Clinical outcomes of transradial vs nontransradial aortoiliac endovascular therapy. JACC Cardiovasc Interv. 2024;17(16):1891–901. 39197987 10.1016/j.jcin.2024.06.002

[6] Mustapha JA, Saab F, McGoff T, Heaney C, Diaz-Sandoval L, Sevensma M, et al. Tibio-pedal arterial minimally invasive retrograde revascularization in patients with advanced peripheral vascular disease: the TAMI technique, original case series. Catheter Cardiovasc Interv. 2014;83(6):987–94. 24214522 10.1002/ccd.25227

[7] Htun WW, Kyaw H, Aung YL, Maw M, Kwan T. Primary retrograde tibio-pedal approach for endovascular intervention of femoropopliteal disease with chronic total occlusion. Cardiovasc Revasc Med. 2020;21(2):171–5. 31699649 10.1016/j.carrev.2019.10.023

[8] Hayakawa N, Kodera S, Hirano S, Arakawa M, Inoguchi Y, Kanda J. An AnteOwl WR intravascular ultrasound-guided parallel wiring technique for chronic total occlusion of below-the-knee arteries. CVIR Endovasc. 2022;5(1):18. 35347485 10.1186/s42155-022-00294-2 PMC8960544

[9] Feldman DN, Armstrong EJ, Aronow HD, Gigliotti OS, Jaff MR, Klein AJ, et al. SCAI consensus guidelines for device selection in femoral-popliteal arterial interventions. Catheter Cardiovasc Interv. 2018;92(1):124–40. 29691970 10.1002/ccd.27635

[10] Tepe G, Laird J, Schneider P, Brodmann M, Krishnan P, Micari A, et al. Drug-coated balloon versus standard percutaneous transluminal angioplasty for the treatment of superficial femoral and popliteal peripheral artery disease: 12-month results from the IN.PACT SFA randomized trial. Circulation. 2015;131(5):495–502. 25472980 10.1161/CIRCULATIONAHA.114.011004 PMC4323569

[11] Ciprian Cacuci A, Krankenberg H, Ingwersen M, Gayed M, Stein SD, Kretzschmar D, et al. Access site complications of peripheral endovascular procedures: a large, prospective registry on predictors and consequences. J Endovasc Ther. 2021;28(5):746–54. 34137662 10.1177/15266028211025044

[12] Rutherford RB, Baker JD, Ernst C, Johnston KW, Porter JM, Ahn S, et al. Recommended standards for reports dealing with lower extremity ischemia: revised version. J Vasc Surg. 1997;26(3):517–38. 9308598 10.1016/s0741-5214(97)70045-4

[13] Steiner S, Schmidt A, Zeller T, Tepe G, Thieme M, Maiwald L, et al. COMPARE: prospective, randomized, non-inferiority trial of high- vs. low-dose paclitaxel drug-coated balloons for femoropopliteal interventions. Eur Heart J. 2020;41(27):2541–52. 31989155 10.1093/eurheartj/ehaa049 PMC7360381

[14] Kuno T, Yamaji K, Aikawa T, Sawano M, Ando T, Numasawa Y, et al. Transradial intervention in dialysis patients undergoing percutaneous coronary intervention: a Japanese nationwide registry study. Eur Heart J Open. 2023;3(6):oead116. 38105921 10.1093/ehjopen/oead116 PMC10721448

[15] Kuno T, Hirano K, Imaeda S, Hashimoto K, Ryuzaki T, Saito T, et al. A transradial approach of cardiac catheterization for patients on dialysis. J Invasive Cardiol. 2018;30(6):212–7. 29335385

[16] Steiner S, Schmidt A. Repeat BTK revascularization: when, how and what are the results? J Cardiovasc Surg (Torino). 2021;62(2):118–23. 33635043 10.23736/S0021-9509.21.11679-9

[17] Mori S, Hirano K, Makino K, Honda Y, Tsutsumi M, Sakamoto Y, et al. Feasibility of ultrasound-guided transoccluded radial access for coronary angiography or percutaneous coronary intervention. JACC Cardiovasc Interv. 2020;13(17):2088–90. 32912468 10.1016/j.jcin.2020.06.035

[18] Uyanık SA, Öğüşlü U, Yılmaz B, Çevik H, Atlı E, Gümüş B. Retrograde pedal access via occluded arteries in endovascular treatment of critical limb ischemia. J Vasc Interv Radiol. 2021;32(2):164–72. 33248916 10.1016/j.jvir.2020.08.034

[19] Miwa H, Hayakawa N, Tsuchida Y, Ichihara S, Hirano S, Maruta S, et al. An effective method for percutaneous hemostasis of femoral artery by endovascular balloon occlusion via the occluded dorsal pedalis artery approach in a patient with restricted access site. SAGE Open Med Case Rep. 2025;13:2050313X251364131. 40761364 10.1177/2050313X251364131 PMC12319259

[20] Iwata S, Tan M, Miwa T, Sasaki W, Urasawa K. Vascular and wound healing outcomes after puncture of small or stenotic inframalleolar arteries in patients with chronic limb-threatening ischemia. J Vasc Surg. 2025;82(5):1736-44.e1. 40409432 10.1016/j.jvs.2025.05.028

[21] Siu HK, Schultz E, LeBrun S, Liou M, Kwan TW. Safety of retrograde tibial-pedal access and intervention in patients with single remaining non-occluded infra-popliteal runoff artery. J Cardiovasc Dev Dis. 2023. 10.3390/jcdd10110463. 37998521 10.3390/jcdd10110463 PMC10672062

